# Generation of human scFvs antibodies recognizing a prion protein epitope expressed on the surface of human lymphoblastoid cells

**DOI:** 10.1186/1472-6750-7-38

**Published:** 2007-07-02

**Authors:** Michela Flego, Alessandro Ascione, Silvia Zamboni, Maria L Dupuis, Valentina Imperiale, Maurizio Cianfriglia

**Affiliations:** 1 Section of Pharmacogenetics, Drug Resistance and Experimental Therapeutics, Department of Drug Research and Evaluation, Istituto Superiore di Sanità, Viale Regina Elena 299, 00161 Rome, Italy

## Abstract

**Background:**

A hallmark of prion disease is the transformation of normal cellular prion protein (PrPc) into an infectious disease-associated isoform, (PrPsc). Anti-prion protein monoclonal antibodies are invaluable for structure-function studies of PrP molecules. Furthermore recent *in vitro *and *in vivo *studies indicate that anti-PrP monoclonal antibodies can prevent the incorporation of PrPc into propagating prions.

In the present article, we show two new human phage antibodies, isolated on recombinant hamster prion protein (rHaPrP).

**Results:**

We adopted an antibody phage display strategy to isolate specific human antibodies directed towards rHaPrP which has been used as a bait for panning the synthetic ETH-2 antibody phage library. Two phage antibodies clones named MA3.B4 and MA3.G3 were isolated and characterized under genetic biochemical and immunocytochemical aspects. The clones were found to recognize the prion protein in ELISA studies. In flow-cytometry studies, these human single chain Fragment variable (scFv) phage-antibodies show a well defined pattern of reactivity on human lymphoblastoid and myeloid cells.

**Conclusion:**

Sequence analysis of the gene encoding for the antibody fragments and antigen recognition patterns determined by flow-cytometry analysis indicate that the isolated scFvs recognize novel epitopes in the PrPc molecule. These new anti PrPc human antibodies are unique reagents for prion protein detection and may represent a biologic platform to develop new reagents to treat PrPsc associated disease.

## Background

The disease-associated PrPsc or transmissible spongiform encephalopathies (TSEs), are invariably lethal neurodegenerative illnesses that affect humans and many animal species; they include bovine spongiform encephalopathy of cattle and Creutzfeldt-Jakob disease (CJD) in humans [1,2]. The causative agent is termed prion and was proposed to be identical to PrPsc, a pathological conformer of the PrPc encoded by the *Prnp *gene [1]. The conversion of the normal PrPc into the abnormal PrPsc isoform is a key feature of prion diseases [3]. Although the molecular mechanisms of conversion are not fully understood, it is known that mature PrPc expressed on the cell surface is essential for prion propagation and pathogenesis. Conversion of PrPc to PrPsc is believed to involve direct interaction of the two prion protein (PrP) isoforms [3,4]. Several agents including anti-PrP monoclonal antibodies (mAbs) have been directed at the binding of the two PrP isoforms to inhibit the conversion of PrPc to PrPsc and ultimately block the neuronal pathogenicity [5,6]. However, the administration of monoclonal antibodies (mAb) generated via hybridoma technology while feasible and effective present several limitations [7]. The 145–150 kDa IgG protein is poorly diffused from vessels into tissues, particularly into the central nervous tissue. This may explain why administration of mAbs has been shown to prevent prion pathogenesis only when administred simultaneously or shortly after peripheral prion infection [6]. It has been also reported that intracerebral injection of anti-PrP IgG antibodies provoked neurotoxicity by cross-linking PrPc [8]. Moreover, the treatment of human patients with rodent monoclonal antibodies is limited by the severe adverse effects due to its xenogenic origin [7]. Recombinant human antibody fragments, may represent an effective alternative for immunotherapy of TSEs [9]. Recently, by applying a biopanning-based approach, we were able to select from the ETH-2 library human scFv phage antibodies specifically recognizing the pathological isoform of the hamster prion protein showing transcurable affinity for the PrPc expressed on human cells [10].

In the present article, we describe new reactive human phage antibodies with a well defined pattern of reactivity on human cell lines. These phage antibodies were isolated using an identical bioapanning-based strategy with rHaPrP as a bait.

The antibody fragments retain the targeting specificity of the whole IgG mAbs but can be produced less expensively and possess other unique and superior properties for diagnostic and therapeutic applications [11].

## Results and Discussion

### Phage antibody selection

**Figure 1:**
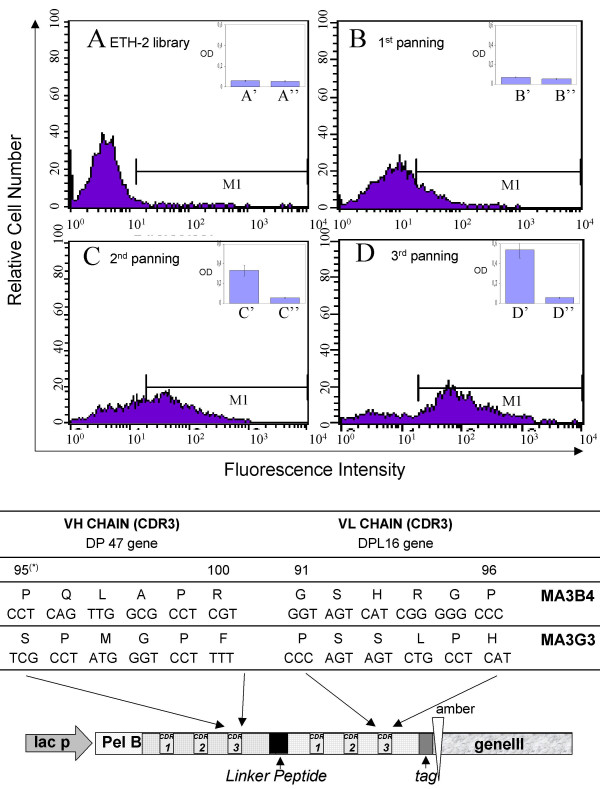
**Phage antibody selection and molecular genetics characterization**. In the upper part of the figure (panels A, B, C and D) the FACS binding profiles of the ETH-2 library and selected phage antibody populations with CCRF-CEM cells are shown after each round of biopanning selection. In the inserted boxes, the ELISA reactivity of the ETH-2 library (A') and selected phage antibody populations (B', C', D') with the rHaPrP are shown in parallel with the irrelevant phage antibody anti glucose oxidase and anti tetanus toxoid (A", B", C" and D"). Experiments were repeated at least twice and mean ± SD from representative experiments (triplicate samples) is shown. The cut-off value separating positive from negative sample was calculated as 3 standard deviation above the mean of the value obtained from irrelevant phage antibodies (0,085 OD). In the lower part of the figure, the nucleotide composition and the corresponding amino acid sequences in the CDR3 regions of the selected scFv antibodies MA3.B4 and MA3.G3 are shown. A schematic representation of the scFv antibody gene as M13 pIII fusion protein is also illustrated.

To isolate phage antibodies specific for PrP protein, an aliquot of the human synthetic ETH-2 library containing approximately 1 × 10^12 ^cfu phages was introduced for panning into Maxisorp immunotubes coated with rHaPrP. Nonspecifically absorbed phages were removed by intensive washing. Specific bound phages were eluted, amplified and used for next round of selection as described [12]. The isolated phage populations were tested in ELISA and flow-cytometry after each step of biopanning. Figure 1 shows that the binding level of polyclonal phage antibodies with rHaPrP and living/intact CCRF-CEM cells parallels with the progression of biopanning selections.

### Phage antibody characterization

**Figure 2:**
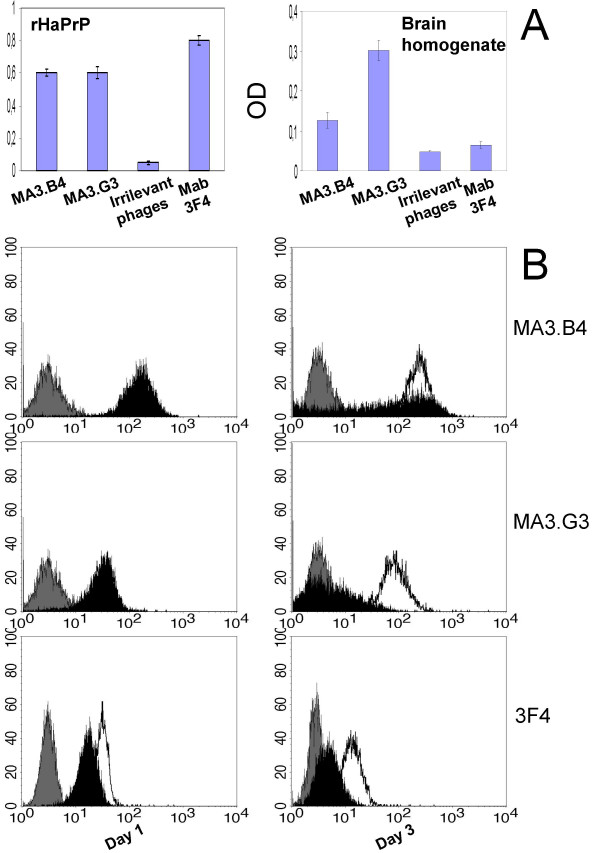
**Phage antibody specificity**. In the panel A, the monoclonal phage antibodies MA3.G3 and MA3.B4 are analyzed by ELISA for rHaPrP and brain homogenates recognition. As controls the irrelevant phage antibody anti glucose oxidase and anti tetanus toxoid are also shown. Experiments were repeated at least twice and mean ± SD from representative experiments (triplicate samples) is shown. The cut-off value separating positive from negative sample was calculated as 3 standard deviation above the mean of the value obtained from irrelevant phage antibodies (0,086 OD for rHaPrp, 0,052 OD for brain homogenate). In panel B, the monoclonal phage antibodies and the rodent mAb 3F4 were tested in flow-cytometry on human HL-60 cells. The surface PrPc expression was down-regulated by ATRA inducing granulocytic differentiation. HL-60 cells were cultured in the presence of 1 μM ATRA for 1 and 3 days, then stained for flow cytometry analysis with scFvs MA3.B4, MA3.G3 and mAb 3F4. Basal level of PrPc staining in undifferentiated cells (solid line) is shown relative to an irrelevant phage antibody, specific for glucose oxidase, or an IgG2b isotype control (in gray). The progressive decrease of PrPc staining during differentiation is shown in black.

After the third round of panning, polyclonal phage antibodies were amplified in TG1 E. coli bacteria and single colonies were picked-up and tested for rHaPrP recognition in ELISA. Two of the most reactive clones named MA3.B4 and MA3.G3 were isolated and further characterized. Molecular-genetics analysis shows that the scFvs MA3.G3 and MA3.B4 displayed on M13 phage are encoded by two different CDR3 sequences (Fig. 1) and that scFvs displayed on phages are intact molecules of 27 kDa (data not shown). Therefore, we tested the PrPc specificity of the phage antibodies verifying their reactivity with hamster brain omogenates in parallel with the irrelevant phage antibodies anti glucose oxidase and anti tetanus toxoid. As shown in figure 2A, the phage antibodies MA3.B4 and MA3.G3 both react with rHaPrp and hamster brain homogenates.

To eliminate concerns about specificity of the phage antibodies we utilized the cell-type specific extinction of PrPc in granulocyte differentiation that can be recapitulated in vitro by *all-trans *retinoic acid (ATRA) treatment of HL-60 cells [13]. Flow-cytometry was performed in order to assess a possible relationship between the down-regulation of surface PrPc and antibody staining. HL-60 cells were treated with ATRA for 72 hrs and stained either with the phage antibodies or with the rodent mAb 3F4. The figure 2B shows a decrease in membrane of prion protein expression in HL-60 cells treated with ATRA *vs *untreated cells when staining is performed with the mAb 3F4 or the phage antibodies MA3.B4 and MA3.G3.

### PrPc typing on human cells

**Figure 3:**
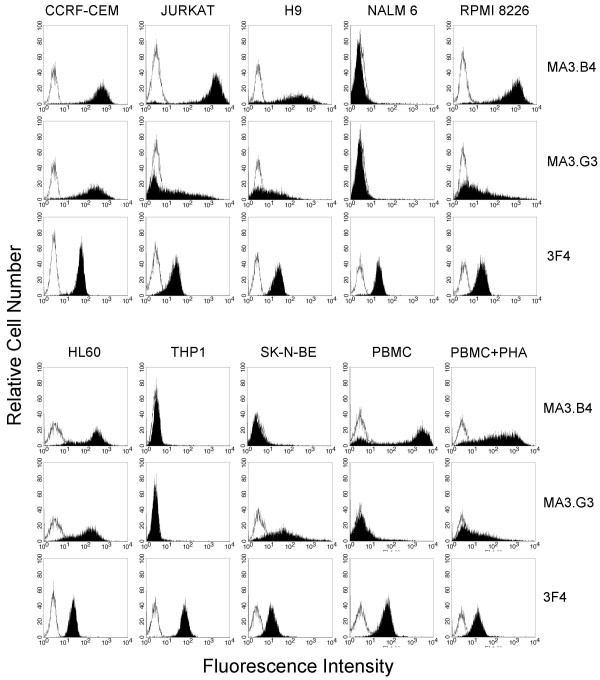
**Flow-cytometry detection**. High representative flow-cytometry profiles of the phage antibodies MA3.G3, MA3.B4 and mAb 3F4 (in black) with human cells of lymphoblastoid, myeloid and neuronal origin are shown in respect to an irrelevant phage antibody, specific for glucose oxidase, or to an IgG2b isotype control (solid line).

PrPc expression was examined with phage antibodies and mAb 3F4 on numerous human cell lines which include lymphobastoid and myeloid cells, the neuroblastoma cell line SK-N-BE, and resting or activated PBMC. As it is shown in figure 3, the staining of the phage antibodies MA3.B4 and MA3.G3 show a well defined reactivity pattern.

The phage antibodies are reactive on CCRF-CEM, Jurkat, H9, RPMI 8226, and HL60 cell lines. When compared with the Mab 3F4, the binding of phage antibodies is significantly different in Nalm-6 and THP-1 cell lines, that react poorly or not at all with the phage antibodies. The phage antibody MA3.B4 is unable to detect PrPc on the SK-N-BE cell line. Furthermore the two phage antibodies show different reactivity on resting and activated human PBMC. Particularly MA3.G3 shows increased staining on PHA-activated human PBMC in respect to resting PBMC according to results previously reported [14].

These pattern of immunoreactivity may be due to the high molecular heterogeneity of PrPc that only for some epitopes may be mimicked by the three dimensional structure of the recombinant PrP expressed by E. coli. Infact, extensive biochemical characterizations conducted with panel of mAbs have demonstrated the molecular complexity of PrPc proteins. Over 50 biochemical forms each representing a distinct PrPc species based on combinations of different molecular weights and isoelectric points (pIs) have been identified by 2-D immunoblot spots [15]. Moreover previously reported data have shown that monoclonal antibodies raised against recombinant human prion protein folded into alpha or beta conformations exhibit striking heterogeneity in their specificity for truncations and glycoforms of mouse brain PrPc [16]. So It may be that one epitopes not present or buried on SK-N-BE, THP1 and Nalm 6 cell lines has been intercepted by the phage antibody MA3.B4 or MA3.G3 selected with rHaPrP as a bait.

**Figure 4:**
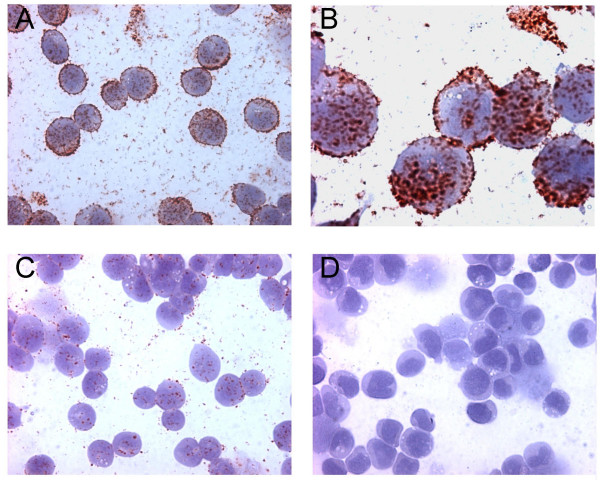
**Immunocytochemical detection**. The immunocytochemical detection of PrP on CCRF-CEM cells with the phage antibody MA3.B4 is shown. In A and B the immunostaining at two different magnification, in C and D the reactivity of CCRF-CEM cells with two distinct irrelevant phage antibodies (anti glucose oxidase and anti tetanus toxoid, respectively) are shown.

Collectively these data suggest that the two phage antibodies do not recognize the same epitope when compared with the rodent mAb 3F4, and the binding properties of the newly isolated scFvs appear to be novel. Furthermore the phage antibodies do not react in Western blot analysis conducted with various prion protein preparations, probably for conformational structure of the target epitope (data not shown). Finally, MA3.B4 phage antibody appears very suitable in PrPc detection in immunocytochemical analysis (Fig. 4).

By summing up our data we can conclude that the human antibodies we isolated and characterized, are the first human phage antibodies in scFv format recognizing the prion protein expressed on the cell membrane of human cells. In fact, previously characterized human phage antibodies have not been tested on prion proteins expressed by viable human cells [17]. Furthermore phage antibodies isolated using the pathological isoform of the hamster prion protein as a bait do not react in ELISA with rHaPrP, and show transcurable reactivity with PrPc on CCRF-CEM cells, indicating that the two classes of phage antibodies recognize distinct epitopes in the prion protein [10].

## Conclusion

Several reports indicate that humoral immune responses against the prion protein can antagonize prion infections [6,18,19]. This is true even when such responses are directed primarily against PrPc and do not selectively target the disease-associated prion protein. Hence, the prospect of immunotherapy against prion diseases has received considerable attention in recent years [20]. To this regard, mAbs specifically recognizing prion protein epitopes expressed on the cell surface of lymphoblastoid cells may represent a valuable reagent for developing anti TSE's therapies. The administration of rodent monoclonal antibodies generated via hybridoma technology [21] while feasible and effective presents several limitations: for example the severe adverse effects due to its xenogenic origin. For these reasons rodent monoclonal antibodies require a technically challenging and expensive genetic manipulation to generate chimeric and/or humanized mAbs [7]. Recombinant antibody fragments, for example scFv antibodies may represent an effective alternative. The antibody fragments retain the targeting specificity of whole mAbs but can be produced more economically and possess other unique and superior properties for diagnostic and therapeutic applications [11].

Data here reported and discussed indicated that the phage antibodies isolated and characterized by us, represent useful reagents for structure/function studies of the PrPc molecule and for developing an immunotherapeutic strategy against prion disease. Furthermore, the genes encoding for the scfvs MA3.B4 and MA3.G3 antibodies have been isolated and sequenced, thus facilitating the delivery of scFvs by genetic transduction *in vivo *for sustained production of scFvs at predefined sites for prolonged periods of time [9].

## Methods

### Prion proteins

Recombinant hamster prion protein, corresponding to the mature form of hamster PrP containing five octarepeats, has been purchased from Prionics AG (Zurich, Switzerland). Homogenates from health hamster were prepared starting from about 2 grams of brain tissue disrupted in a Dounce homogenizer with 9 volumes of PBS/0.5 % Nonidet P-40/0.5 % sodium deoxycholate. Insoluble debris was removed by centrifugation at 1650 rpm for 20 minutes at 4°C. The supernatants were aliquoted and stored at -80°C.

### ETH-2 library

The ETH-2 synthetic human recombinant antibodies library consists of a large array (more than 10^9 ^antibody combination) of scFv polypeptides displayed on the surface of M13 phage. It was built by random mutagenesis of the CDR3 of only three antibody germline gene segments (DP47 for the heavy chain, DPK22 and DPL16 for the light chain). Diversity of the heavy chain was created by randomizing four to six positions replacing the pre-existing positions 95 to 98 of the CDR3. The diversity of the light chain was created by randomizing six positions (96 to 101) in the CDR3 [22].

### Selection of prion protein specific phage antibodies from ETH-2 library

Immunotubes were coated overnight (ON) at room temperature (RT) with rHaPrP in PBS at the concentration of 10 μg/ml. After panning, performed according with Ascione et al. [12] phages were eluted with 1 ml of 100 mM triethylamine and the solution was immediately neutralized by adding 0.5 ml of 1 M Tris-HCl pH 7.4. Eluted phages were used to infect log phase TG1 *E. coli *bacteria and amplified for the next round of panning. Briefly, 50 ml of 2xTY with 100 μg/ml ampicillin and 1% glucose (2xTY-amp-glu) were inoculated with enough bacterial suspension to yield an OD_600 nm_≅ 0.05–0.1. The culture was grown to OD_600 nm _0.4–0.5 and infected with K07 helper phage in a ratio of around 20 : 1 phage/bacteria. The rescued phages were concentrated by precipitation with PEG 6000 and used for next round of panning. For monoclonal phage antibodies preparation, individual TG1 bacterial colonies harbouring phagemides were inoculated in 150 μl 2xTY-amp-glu in 96 well plates, incubated for 2 hours at 37°C and reinfected with 10^9 ^cfu K07 helper phage in 25 μl 2xTY. After 30 minutes, plates were centrifuged at 1800 g for 10 minutes and bacterial pellet resuspended in 200 μl 2xTY with 100 μg/ml ampicillin and 25 μg/ml kanamycin (2xTY-amp-kan). The following day the plates were spinned at 1800 g for 10 minutes and the supernatants containing phage antibody were recovered and tested in ELISA.

### ELISA

96 well ELISA-plates were coated ON with 0.5 μg of rHaPrP in PBS or 5 μl of homogenated brain extracts (from healthy hamsters) diluted in 50 μl of PBS ON at RT. Next day a blocking solution consisting of PBS with 2% non-fat dry milk (2% MPBS) was added; plates were washed with PBS containing 0.05% Tween 20 (TPBS) and incubated for 1 hour at RT with phage supernatants. Plates were washed and incubated for 1 hour with HRP mouse anti-phage antibody (Amersham) resuspended in 2% MPBS. The reaction was developed using 3,3^1^-5,5^1^-tetramethylbenzidin BM blue, POD-substrate soluble (Roche Diagnostics; IN, USA) and stopped by adding 50 μl of 1 M sulfidric acid. The reaction was detected with an ELISA reader (BIORAD; CA, USA), and the results were expressed as A (absorbance) = A(450 nm)-A(620 nm).

### Cells

CCRF-CEM is a human T acute lymphoblastic leukaemia cell line. Jurkat is a human T acute leukemia cell line. RPMI 8226 is a human B Myeloma cell line. Nalm-6 is a human pre-B acute lymphoblastic leukemia cell line. HL60 is a human, acute promyelocytic leukemia, cell line. THP1 is a human, acute monocytic leukemia, cell line. H9 is a human T acute lymphoblastic leukemia cell line. SK-N-BE is a human neuroblastoma cell line.

Cells were cultured in RPMI-1640 supplemented with 10% foetal calf serum (FCS), L-glutamine (2 mM) penicillin (100 U/mL) and streptomycin (100 U/mL), all of which were purchased from Hyclone (Logan, UT). Regulation of prion protein expression in HL60 cell line was induced by culture in 1 μmol ATRA (Sigma) for 1 and 3 days. Cells were then stained for flow cytometry.

PBMC were separated from heparinized venous blood from healthy donors by centrifugation (20 min, 600 *g*) over Ficoll (Gibco) and activated by phytohemagglutinin P (PHA-P, 1 μg/ml, Sigma) for 4 days that stimulates T lymphocytes.

### Flow cytometry determination of PrPc

The expression of PrPc on cells surface was determined by flow-cytometry studies. Cells in exponential phase of growth were collected, extensively washed in PBS and pelletted. About 5 × 10^5 ^cells were resuspended with 50 μl 2% MPB containing ≈ 10^12 ^cfu phage particles and incubated for 1 hour RT. After several washings cells were resuspended for 1/2 hour RT in 2% MPBS solution containing 10 μg/ml of mouse M13 secondary antibody (Amersham), and after washings, cells were again incubated with 6 μg/ml FITC-goat anti mouse Ig (Pierce, IL USA) for 1/2 hour at 4°C. Controls include irrelevant phage antibodies directed to glucose oxidase and to tetanus toxin, an IgG2b isotype control and mAb 3F4 (Dako).

After staining cell samples were washed, maintained at 4°C and immediately analyzed by FACS can (Becton-Dickinson, NJ USA) equipped with 15 nW argon laser. Fluorescence compensation was determined using samples stained with mouse M13 and FITC-conjugated goat anti mouse secondary antibodies.

All experiments were done at least two times.

### Immunocytochemistry

For cytospin preparation, 3 × 10^5 ^CCRF-CEM cells for slide were spinned at 600 rpm in cytospeen 3 Shandom centrifuge (Pittsburgh PA USA) for 5 minutes and immediately immersed in 80 % methanol solution for 10 minutes at 4°C. After three 5 minutes washes, in TBS, the slides were treated for ten minutes with peroxidase block solution from Dako En Vision system HRP (AEC), washed and blocked with RPMI and 10% FCS (both from Hyclone Laboratories) 10 minutes. The slides were incubated for 1 hour at RT in presence of 10^11 ^cfu phage particles, extensively washed and incubated with mouse M13 secondary antibody (Amersham) for 1 hour RT. Finally treated with labelled polymer solution 30 minutes, and then with AEC substrate cromogen both belonging to the KIT. The counter stain was performed with mayer haematoxylin. Each incubation was followed by three washes in TBS.

### DNA characterization and sequences

Plasmid DNA from individual bacterial colonies of MA3.B4 and MA3.G3 clones was digested with specific endonucleases and CDR3 regions were sequenced with an automated DNA sequencer (M-Medical/Genenco, Pomezia Italy) using fdseq1 (5'-GAA TTT TCT GTA TGA GG-3') and pelBback (5'-AGC CGC TGG ATT GTT ATT AC-3') primers.

## Abbreviations

ATRA, all-trans retinoic acid; CDR, complementarity determining regions; CJD, Creutzfeldt-Jacob disease; ETH-2, synthetic scFv antibody phage library; mAbs, monoclonal antibodies. PrP, prion protein; PrPc, cellular prion protein; PrPsc, disease associated prion protein; rHaPrp, recombinant hamster prion protein; scFv, single chain fragment variable; TSE, transmissible spongiform encephalopaties;

## Competing interests

The author(s) declare that they have no competing interests.

## Authors' contributions

MF carried out phage antibody selection, participated in the genetic molecular, biochemical and Immunocytochemical characterization of phage antibody specific for PrP.

AA participated to phage antibodies selection and carried out immunoassay and biochemical characterization of the antibodies to PrP.

MLD carried out all the flow-cytometry determinations of phage antibody binding on cells

VI participated to genetic and biochemical characterization of phage antibodies to PrP.

SZ participated to genetic and biochemical characterization of phage antibodies to PrP.

MC conceived of the study, promotes the approach with phage library to select specific phage antibodies against recombinant PrP protein. Furthermore, MC designed and coordinated the research and drafted the manuscript.

All authors have read and approved the final version of the manuscript.
